# Phase Angle as a Comprehensive Tool for Nutritional Monitoring and Management in Patients with Crohn’s Disease

**DOI:** 10.3390/nu14112260

**Published:** 2022-05-28

**Authors:** Ziheng Peng, Duo Xu, Yong Li, Yu Peng, Xiaowei Liu

**Affiliations:** 1 Department of Gastroenterology, Xiangya Hospital of Central South University, Changsha 410008, China; pengzh96@csu.edu.cn (Z.P.); xuduo1997@csu.edu.cn (D.X.); stanlee@csu.edu.cn (Y.L.); 2 Hunan International Scientific and Technological Cooperation Base of Artificial Intelligence Computer Aided Diagnosis and Treatment for Digestive Disease, Changsha 410008, China; 3 National Clinical Research Center for Geriatric Disorders, Xiangya Hospital, Central South University, Changsha 410008, China

**Keywords:** phase angle, Crohn’s disease, GLIM, nutritional status, nutritional monitoring and management

## Abstract

Background and Aims: Crohn’s disease (CD) is usually accompanied by malnutrition. CD-related malnutrition can increase morbidity, disability, mortality, and hospitalization costs. The purpose of this study was to find a reliable indicator for evaluating CD patients’ nutritional status. Methods: All data were retrospectively collected from Xiangya Hospital, Central South University between May 2021 and February 2022. All patients were evaluated for nutritional status using the Global Leadership Initiative on Malnutrition (GLIM) criteria. Body composition, resistance, and reactance were recorded by a body analyser, and the phase angle (PhA) was calculated simultaneously. The Mann–Whitney U test, chi-square test, Fisher’s exact test, and univariate and multivariate logistic regression analyses were used. A receiver operating characteristic (ROC) curve was built to evaluate the predictive value of differential variables for diagnosing malnutrition based on the GLIM criteria. Results: A total of 169 CD patients were enrolled, of which 74 (58.3%) males and 32 (76.2%) females were diagnosed with malnutrition; 34 (45.9%) males and 22 (68.8%) females were severely malnourished. Univariate analysis identified that as nutritional status deteriorated, body mass index, PhA, and levels of haemoglobin and albumin decreased, while platelet count, erythrocyte sedimentation rate, and levels of C-reactive protein and fibrinogen increased (*p* < 0.05). Logistic regression analysis revealed that the PhA was significantly independently associated with malnutrition (*p* < 0.05). The ROC curve analysis indicated that the optimal PhA cut-off levels of 6.11° and 5.55° could be used to predict malnutrition according to the GLIM criteria in males and females, respectively, with a PhA < 5.53° and < 5.12° indicating severe malnutrition in males and females, respectively. Conclusion: The PhA is a sensitive, noninvasive, portable, inexpensive tool that can be used to monitor and manage the nutritional status of CD patients.

## 1. Introduction

Crohn’s disease (CD), a type of inflammatory bowel disease (IBD), is a nonspecific chronic intestinal inflammatory disorder of unknown aetiology. Heredity, immunology, and environment are potential risk factors. The epidemiology of CD is changing, and the prevalence of CD has steadily increased in Western countries over the past several decades as well as among previously low-incidence non-White races and ethnicities. [1,2] As many as 80% of CD patients experience malnutrition [3,4]. Several factors may lead to malnutrition in CD patients, including reduced oral food intake, bacterial overgrowth, impaired epithelial transport, and loss of epithelial integrity [4]. Disease-related malnutrition is a syndrome associated with substantially increased morbidity, disability, mortality, and hospitalization costs [5]. Notably, a large retrospective study has found that malnutrition related to IBD is associated with increased in-hospital mortality rate (odds ratio [OR]:3.49, 95% confidence interval [CI]: 2.89–4.23), length of stay (11.9 days versus 5.8 days, *p* < 0.00001), and total charges (USD 45,188 versus USD 20,295, *p* < 0.0001) [6]. Hence, it is important for physicians and patients to identify and address malnutrition as early as possible.

Considered to be a new promising nutritional status assessment method, bioelectrical impedance analysis (BIA) is a noninvasive tool that can be used to assess body composition based on the relationship between total body impedance and total body water. The phase angle (PhA), which represents an indicator of cellular health and membrane integrity, is a variable that is easily calculated using resistance (R) and reactance (Xc) values determined by BIA [7]. Recently, several studies have indicated that the BIA-derived PhA, a superior prognostic marker, can be considered as a screening tool for the identification of at-risk patients with impaired nutritional and functional status [8]. There is growing interest in nutritional monitoring and management based on the PhA in patients with several diseases, such as heart failure, obesity, chronic obstructive pulmonary disease, and hip fractures [9,10,11,12,13].

There have been previous attempts to understand the PhA and nutritional status of CD patients. In a limited number of available studies, the PhA has been found to be different in CD patients with different nutritional statuses. However, studies attempting to assess PhA in order to identify and monitor nutritional risk in CD patients have not yielded consistent results. The PhA has been reported as a valid indicator of nutritional status in adults with CD [14] and patients on infliximab therapy [15]; however, other studies have concluded the opposite. Furthermore, the PhA was not found to be associated with nutritional status in paediatric IBD patients [16] or in another cohort undergoing infliximab therapy [17]. Consequently, the relationships between PhA and CD remain unclear.

The main purpose of this work was to explicitly evaluate the role that the PhA plays in the nutritional status of CD patients and to preliminarily explore whether the PhA could be used as a tool for nutritional monitoring and management.

## 2. Materials and Methods

### 2.1. Patients and Design

Data from CD patients were collected retrospectively from Xiangya Hospital, Central South University, between May 2021 and February 2022. All CD patients met the following criteria: (1) age ranging from 18–65; (2) diagnosed with CD at the IBD Center; and (3) willing to undergo BIA. The exclusion criteria were as follows: (1) inability to finish the BIA or incomplete BIA data; (2) malignancy related to CD or other malignant tumours; and (3) other factors that may lead to malnutrition, such as anorexia nervosa and dysphagia.

### 2.2. Clinical Measurements

Participants’ age, sex, and BIA variables were collected. The disease activity was collected and calculated using the Crohn’s disease activity index (CDAI). Remission is commonly defined as a CDAI of ≤150, while activity is defined as a CDAI > 150 [18]. In addition, the age of diagnosis, lesion locations, and disease behaviour of CD, presence of perianal disease, and history of gastrointestinal surgery were recorded. Patient laboratory variables, including the haemoglobin (HGB) level, white blood cell (WBC) and platelet (PLT) counts, albumin (ALB) level, coagulation function, erythrocyte sedimentation rate (ESR), and C-reactive protein (CRP) levels were retrospectively reviewed and collected from electronic medical records.

### 2.3. BIA, BMI, FFMI, and PhA

BIA is a safe and noninvasive method for measuring the electrical characteristics of subjects and was performed in accordance with the manufacturer’s guidelines. [9,19,20] Patients stepped into the body composition analyzer in a standing position, held the handles on both sides, and waited for an ‘end’ beep, which occurred after 10 s. Using BIA data, the fat-free mass index (FFMI) and body mass index (BMI) can be calculated quickly [21]. Bioelectrical resistance (R, ohm) and reactance (Xc, ohm) were obtained with the phase-sensitive 50 kHz impedance device (Tanita, MC-180, Tokyo, Japan) of the BIA system. The PhA was recorded as the arctangent of the Xc/R ratio, and the specific formula was defined as follows: [Xc(RH+RL)/R(RH+RL)] × 180°/π [22].

### 2.4. Malnutrition Diagnosis

The patients’ nutritional status was identified following to the recommendations of the Global Leadership Initiative on Malnutrition (GLIM). The diagnosis of malnutrition requires at least 1 of 3 phenotypic criteria (nonvolitional weight loss, low BMI, and reduced muscle mass) and 1 of 2 aetiological criteria (reduced food intake/assimilation and inflammation/disease burden) [23]. In our study, all patients were considered to meet the aetiological criteria in the presence of disease. Patients were categorized into groups on the basis of the presence or absence of malnutrition following the GLIM criteria. Furthermore, BMI values of 20 kg/m^2^ and 18.5 kg/m^2^ or weight loss percentages of 5% and 10% in the past six months were used as the cut-off values below which undernourished patients were classified as having moderate or severe malnutrition, respectively.

### 2.5. Statistical Analysis

Statistical analyses were performed using SPSS for Mac OS X version 23 (SPSS Inc., Chicago, IL, USA). First, descriptive analyses were conducted in order to determine whether the data were normally distributed. Values are presented as the mean and standard deviation (SD) for normally distributed data or as the median (interquartile range) for non-normally distributed data. Second, the significance of differences among groups was evaluated using the Mann–Whitney U test for numerical variables and the chi-square or Fisher’s exact test for categorical data. Third, univariate and multivariate logistic regression analyses were performed in order to further identify differential variables. Of note, BMI was excluded because it is considered in the GLIM criteria. Finally, a receiver operating characteristic (ROC) curve was built to evaluate the predictive value of differential variables for malnutrition diagnosis based on the GLIM criteria. A *p*-value ≤0.05 was considered statistically significant for all analyses.

## 3. Results

A total of 169 CD patients were included in this cross-sectional study. Of these, 127 patients (75.1%) were males, with an age of 30.87 ± 11.05 years, and 42 (24.9%) were females, with an age of 31.10 ± 12.39 years. The BMI was 19.93 ± 3.38 kg/m^2^ and 18.93 ± 2.94 kg/m^2^ in males and females, respectively. The PhA was 6.01 ± 0.75° in males and 5.28 ± 0.76° in females. The Montreal classification was used to define the diagnosis age, lesion range, and disease behaviour [24]. Other laboratory examination results and disease characteristics are summarized in Table 1.

**Figure 1:**
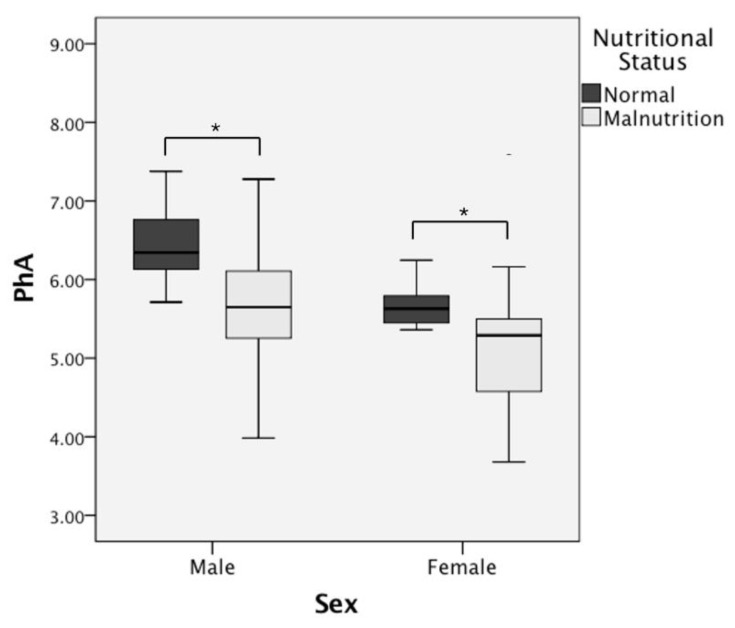
Box plot comparing PhA in CD patients with different nutritional statuses. Note: *: *p* < 0.05.

The patients were divided into two groups, i.e., the malnourished and nourished groups, based on the GLIM criteria (Table 2): 74 (58.3%) males were malnourished, while 32 (76.2%) females were malnourished. When comparing the two groups, in both males and females the BMI and PhA of malnourished patients were significantly lower than those of nourished patients. Differences in the PhA of CD patients with different nutritional statuses are shown in a box plot in Figure 1.

Moreover, in males the HGB and ALB levels were significantly lower, while the fibrinogen (FIB) level, ESR, and CRP level were significantly greater in the malnourished group than in the nourished group (*p* < 0.05). There was a trend for a higher proportion of malnourished male patients to be in the active stage (*p* = 0.004). However, this phenomenon was not observed in females in our study. No significant differences were found in lesion location, disease behaviour, perianal complications, or history of surgery between the two groups. The PhA (*p* = 0.000) was an independent factor associated with malnutrition in males according to the bivariable logistic regression analysis (Table 3). Because PhA was the only factor to show a significant difference between the two groups of females in the univariate analysis, no further bivariate logistic regression analyses were performed.

**Figure 2:**
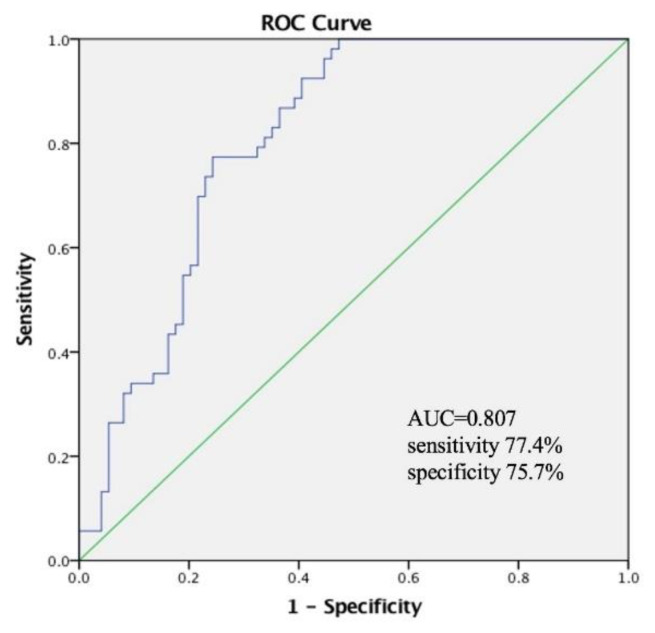
Receiver-operating curve analysis for phase angle (PhA) to diagnose malnutrition in males according to the GLIM criteria with optimal PhA level cut-off of 6.11°.

**Figure 3:**
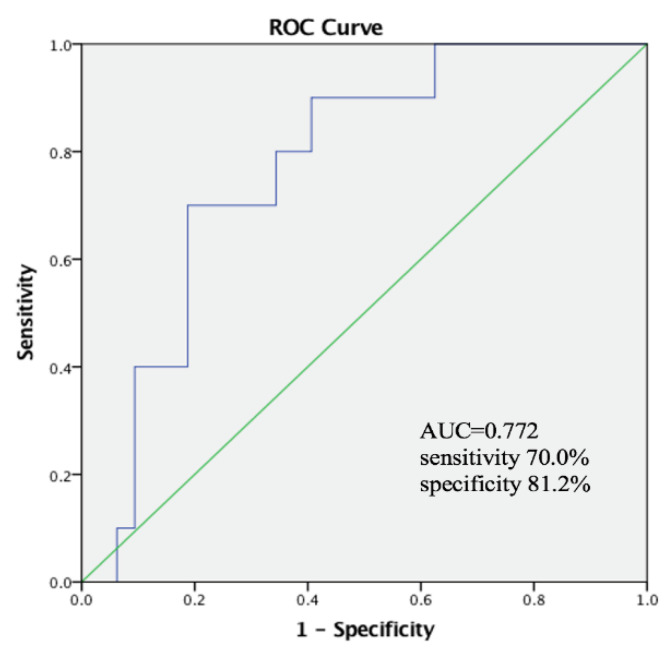
Receiver-operating curve analysis for phase angle (PhA) to diagnose malnutrition in females according to the GLIM criteria with optimal PhA level cut-off of 5.55°.

An ROC curve analysis was performed for malnutrition according to the GLIM criteria. PhA showed good diagnostic accuracy for malnutrition in males (Figure 2). An ROC curve analysis was used to determine the optimal PhA cut-off level of 6.11° for predicting malnutrition according to the GLIM criteria, with an area under the curve (AUC) of 0.807, a sensitivity of 77.4%, and a specificity of 75.7%. Patients with a PhA < 6.11° had a significantly higher rate of malnutrition than those without a PhA < 6.11° (*p* = 0.000). An ROC curve analysis was performed for female patients as well; the optimal PhA cut-off level for predicting malnutrition in females was 5.55°, with an AUC of 0.772, a sensitivity of 70.0%, and a specificity of 81.2% (Figure 3).

**Figure 4:**
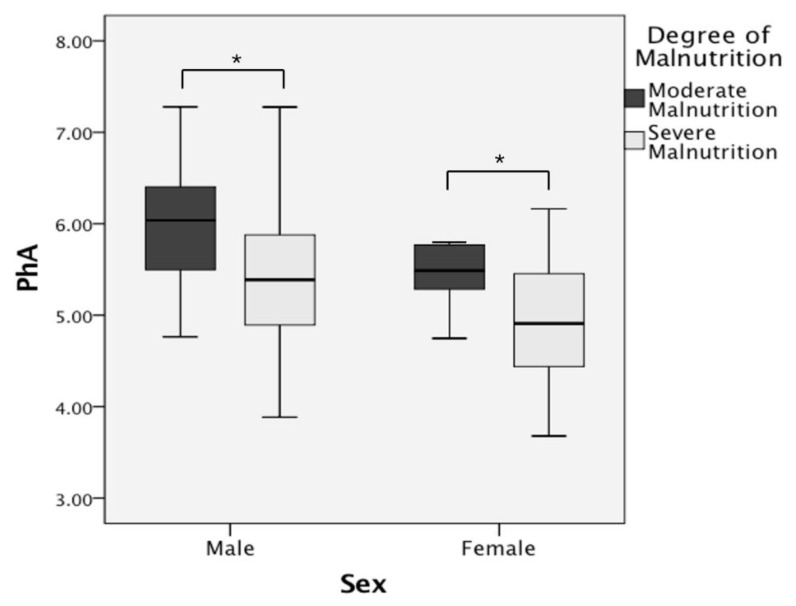
Box plot comparing PhA in CD patients with different degrees of malnutrition. Note: *: *p* < 0.05.

Going a step further, we divided the malnutrition patients into two groups according to their degree of malnutrition based on the GLIM criteria (Table 4); there were 40 (54.1%) patients with moderate malnutrition and 34 (45.9%) patients with severe malnutrition. In both males and females, the BMI, PhA, and HGB and ALB levels decreased as the severity of malnutrition worsened, while the PLT count increased (*p* < 0.05). The FIB level, ESR, and CRP level were significantly higher in males classified as severely undernourished than in those classified as moderately undernourished (*p* < 0.05). The proportion of high disease activity was even higher in severely malnourished male patients (p = 0.003). Interestingly, compared with female patients with moderate malnutrition, female patients with severe malnutrition had a younger age of diagnosis. Differences in the PhA of CD patients with different degrees of malnutrition are shown in a box plot (Figure 4). In the bivariable logistic regression analysis, a lower PhA (*p* = 0.016 & 0.037) was significantly associated with the degree of malnutrition regardless of sex (Table 5 and Table 6).

**Figure 5:**
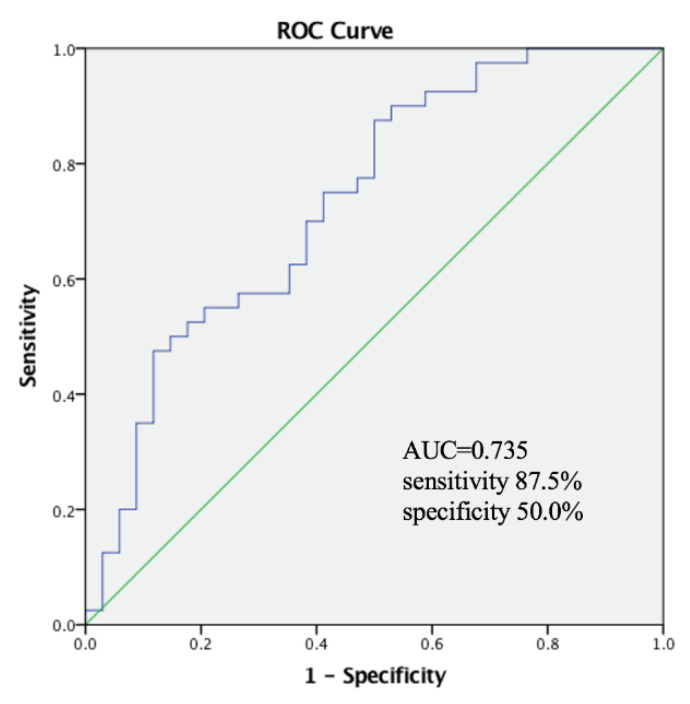
Receiver-operating curve analysis for phase angle (PhA) to distinguish moderate/severe malnutrition in man according to the GLIM criteria with optimal PhA level cut-off of 5.53°.

**Figure 6:**
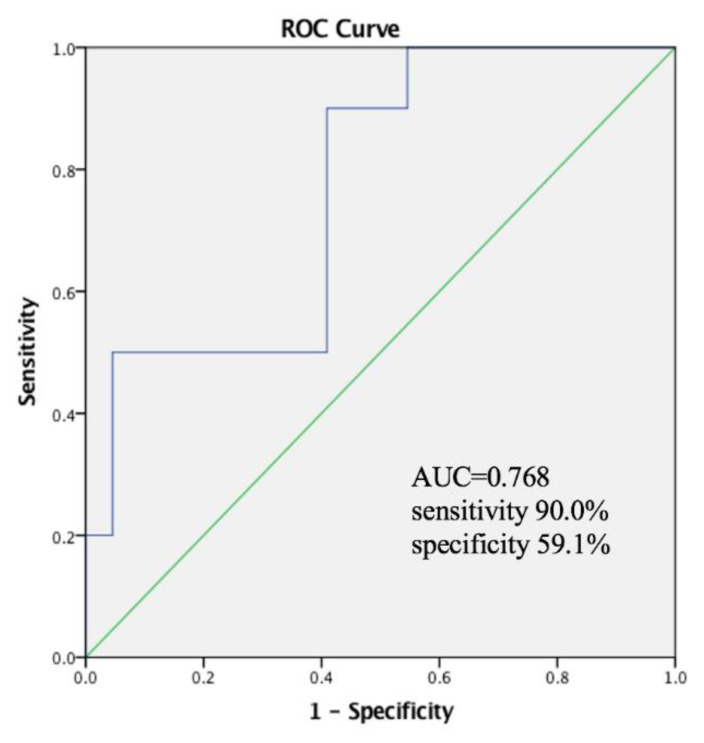
Receiver-operating curve analysis for phase angle (PhA) to distinguish moderate/severe malnutrition in woman according to the GLIM criteria with optimal PhA level cut-off of 5.12°.

The ROC curve analysis indicated that PhA had moderate discriminatory power for differentiating the degree of malnutrition in males, with an AUC of 0.735, a sensitivity of 87.5%, and a sensitivity of 50.0% (Figure 5). Patients with a PhA < 5.53° had a significantly higher rate of severe malnutrition than those with a higher PhA. Among females, those with a PhA < 5.12° had a significantly higher rate of severe malnutrition, with an AUC of 0.768, a sensitivity of 90.0%, and a sensitivity of 59.1% (Figure 6).

## 4. Discussion

In this study, more than half (62.7%) of CD patients were malnourished based on the GLIM criteria, which is similar to the findings of previous studies [25,26]. The current study confirmed the ability of PhA to identify malnourished CD patients as well as to further differentiate between severe and nonsevere malnutrition. PhA could thus be used as an effective tool in nutritional monitoring and management.

In addition, our study showed higher levels of inflammation-related markers such as the PLT count, FIB level, ESR, and CRP level along with greater disease activity in malnourished patients, indicating that the levels of these inflammatory markers are related to nutritional status. Diet might be a key factor in bodily communication regarding nutrition and inflammation. Diet has been shown to be a structural determinant of the gut microbial community [27], which affects the immune system and inflammatory response by altering microbial structure and function as well as by interacting directly with gut mucosal defence and inflammatory cells [28,29,30].

In this study, HGB was related to nutritional status and malnutrition severity in CD patients, which is similar to the findings of previous studies [3,31,32,33]. More than half of IBD patients have been found to be deficient in iron, [34] and chronic wasting and lack of vitamin B_12_ are other aetiologies of anaemia in IBD patients. Two factors might lead to iron deficiency anaemia in patients with CD, namely, dysfunctional iron absorption and iron consumption caused by inflammation [35].

Moreover, in the univariate regression analysis malnourished males were younger than nourished males. Additionally, severe malnutrition was more common at an earlier age in females. Thus, age might affect the inflammatory process and clinical phenotype [36,37]. Compared to elderly patients, younger patients show more severe intestinal invasiveness and are accompanied by more complications [38,39].

The evaluative criteria for malnutrition are incomprehensive, and there is an urgent need to apply a comprehensive objective standard for diagnosing malnutrition. Recently, the GLIM criteria have been adopted as a protocol for diagnosing adult malnutrition in clinical work [23]. The GLIM criteria have been used in several influential studies, and clinical guidelines have been created by researchers [40,41,42,43,44]. The present study combines the PhA with the GLIM criteria for the first time. The PhA can serve as a sensitive indicator for monitoring the nutritional status of CD patients. According to several studies, the PhA is related to the nutritional status of patients in other diseases, with a lower PhA indicating poorer nutritional status [7,45,46,47,48]. These findings are similar to those in our study, which showed a positive correlation between PhA and nutritional status [14,15,49]; PhA < 6.11° and < 5.55° could be used as cut-off points for diagnosing malnutrition in males and females, respectively. Furthermore, PhA could be used to distinguish between severe and nonsevere malnutrition, at a cut-off of 5.53° in males and 5.12° in females.

Through an analysis of weight loss, BMI, and reduced muscle mass, nutrition monitoring according to the GLIM criteria is more precise and objective [50]. Therefore, it is likely that only individuals with professional medical knowledge can make a correct judgement. The PhA relies on BIA for its calculation, and the implementation of BIA is easy, noninvasive, and inexpensive. BIA allows for monitoring of a patient’s nutritional status by reflecting the body’s cellular density, cellular size, and cellular membrane integrity [9,45]. Only one instrument is required for BIA. After simple training, a social worker or patient can obtain an assessment of nutritional status in real time and contact a doctor in a timely manner; after all, CD requires lifelong treatment, and timely nutritional support is beneficial in enhancing drug response rate and decreasing the rate of complications [51,52]. Moreover, because BIA is noninvasive and cost efficient it is easy for patients to accept, and can reduce the economic burden imposed by the disease itself to an extent. [53,54,55] The fairly good discrimination of PhA between different levels of severity in undernourished patients is interesting as well. It can be hypothesized that the PhA might become a comprehensive tool for nutritional monitoring and management among CD patients in the near future.

Certainly, there are limitations to the present study. First, this was a single-centre cross-sectional survey with a small sample size. Second, for both objective and subjective reasons there might be a possibility of bias in this study, and the credibility of the results needs to be further verified by external validation. Third, a more in-depth analysis including more indicators, such as micro-nutritional status, might allow more accurate conclusions to be drawn about the nutritional status of CD patients. However, the current limited research data have yielded conflicting results [56,57]. Further studies involving larger sample sizes, more comprehensive analysis, and an expansion of the research scope are required in order to verify these findings and shed more light on the topic.

## 5. Conclusions

In this study, we evaluated the role of the PhA in assessing the nutritional status of CD patients. The results indicate that the PhA could be a comprehensive tool for nutritional monitoring and management. However, it is unclear whether it is possible to initiate individualized nutritional interventions according to the severity of malnutrition based on PhA discrimination. This possibility needs to be further explored in future multicentre randomized controlled studies.

## Figures and Tables

**Table 1:** Demographic, anthropometric, body composition, and clinical data of CD patients.

Variable	CD Patients (n = 169)	
	Men (n = 127)	Women (n = 42)
**Age (year)**	30.87 ± 11.05	31.10 ± 12.39
**BMI (kg/m^2^)**	19.93 ± 3.38	18.93 ± 2.94
**PhA (°)**	6.01 ± 0.75	5.28 ± 0.76
**WBC (** **×10^9^/L)**	6.24 ± 2.18	5.82 ± 1.68
**HGB (g/L)**	129.51 ± 24.83	108.98 ± 16.86
**PLT (** **×10^9^/L)**	303.62 ± 104.06	318.12 ± 121.63
**ALB (g/L)**	41.18 ± 5.54	38.93 ± 6.29
**FIB (g/L)**	3.79 ± 1.36	3.88 ± 1.15
**ESR (mm/h)**	40.28 ± 32.11	52.14 ± 35.40
**CRP (mg/L)**	18.80 ± 24.85	19.63 ± 27.30
**Disease activity, n (%)**		
**Activity**	42 (33.1%)	14 (33.3%)
**Remission**	85 (66.9%)	28 (66.7%)
**Age of diagnosis, n (%)**		
**A1**	8 (6.3%)	5 (11.9%)
**A2**	86 (67.7%)	30 (71.4%)
**A3**	33 (26.0%)	7 (16.7%)
**Location, n (%)**		
**L1**	26 (20.5%)	5 (11.9%)
**L2**	6 (4.7%)	2 (4.8%)
**L3**	92 (72.4%)	34 (81.0%)
**L4**	3 (2.4%)	1 (2.4%)
**Behavior, n (%)**		
**B1**	64 (50.4%)	19 (45.2%)
**B2**	38 (29.9%)	22 (52.4%)
**B3**	8 (6.3%)	0 (0.0%)
**B2+B3**	17 (13.4%)	1 (2.4%)
**Combined perianal fistulas, n (%)**		
**Yes**	89 (70.1%)	19 (45.2%)
**No**	38 (29.9%)	23 (54.8%)
**History of gastrointestinal surgery, n (%)**		
**Yes**	62 (48.8%)	18 (42.9%)
**No**	65 (51.2%)	24 (57.1%)

Note: Mean ± SD. Abbreviations: CD, Crohn’s disease; BMI, body mass index; PhA, phase angle; WBC, white blood cell; HGB: hemoglobin; PLT, platelet; ALB, albumin; FIB, fibrinogen; ESR, erythrocyte sedimentation rate; CRP, C-reactive protein; A1: below 16 years old; A2: between 17 and 40 years old; A3: the age of diagnosis is above 40 years old; L1: ileal; L2: colonic; L3: ileocolonic; L4: isolated upper disease; B1: non-stricturing, non-penetrating; B2: stricturing; B3: penetrating; B2+B3: structuring and penetrating.

**Table 2:** Comparison of patient characteristics, body composition, and clinical data between adequate nutrition status and malnutrition in CD.

	Men (n = 127)		*p*-Value	Women (n = 42)		*p*-Value
	Normal (n = 53)	Malnutrition (n = 74)		Normal (n = 10)	Malnutrition (n = 32)	
**Age (year)**	32.91 ± 10.59	29.44 ± 11.51	0.025 *	37.50 ± 14.74	28.91 ± 11.05	0.085
**BMI (kg/m^2^)**	22.74 ± 3.28	17.99 ± 2.02	0.000 *	22.93 ± 1.40	17.68 ± 2.01	0.000 *
**PhA (°)**	6.45 ± 0.51	5.65 ± 0.75	0.000 *	5.63 ± 0.36	5.17 ± 0.82	0.010 *
**WBC (** **×10^9^/L)**	6.27 ± 2.09	6.41 ± 2.35	0.584	5.95 ± 0.71	5.78 ± 1.90	0.478
**HGB (g/L)**	134.82 ± 26.74	124.72 ± 22.47	0.009 *	113.60 ± 13.04	107.53 ± 17.82	0.367
**PLT (** **×10^9^/L)**	291.07 ± 105.92	316.59 ± 100.58	0.102	294.50 ± 66.32	325.50 ± 134.36	0.555
**ALB (g/L)**	42.44 ± 4.50	39.88 ± 6.36	0.027 *	40.13 ± 4.50	38.55 ± 6.77	0.525
**FIB (g/L)**	3.49 ± 1.15	4.03 ± 1.52	0.021 *	3.96 ± 0.92	3.86 ± 1.23	0.595
**ESR (mm/h)**	33.61 ± 30.00	44.94 ± 32.97	0.014 *	46.60 ± 30.41	53.88 ± 37.10	0.565
**CRP (mg/L)**	13.48 ± 22.06	25.61 ± 31.81	0.005 *	7.94 ± 5.42	23.28 ± 30.33	0.626
**Disease activity, n (%)**			0.004 *			0.306
**Activity**	10 (18.9%)	32 (43.2%)		2 (20.0%)	12 (37.5%)	
**Remission**	43 (81.1%)	42 (56.8%)		8 (80.0%)	20 (62.5%)	
**Age of diagnosis, n (%)**			0.064			0.075
**A1**	1 (1.9%)	8 (10.8%)		1 (10.0%)	4 (12.5%)	
**A2**	40 (75.5%)	45 (60.8%)		5 (50.0%)	25 (78.1%)	
**A3**	12 (22.6%)	21 (28.4%)		4 (40.0%)	3 (9.4%)	
**Location, n (%)**			0.908			0.192
**L1**	12 (22.6%)	14 (18.9%)		3 (30.0%)	2 (6.3%)	
**L2**	3 (5.7%)	3 (4.1%)		0 (0.0%)	2 (6.3%)	
**L3**	37 (69.8%)	55 (74.3%)		7 (70.0%)	27 (84.4%)	
**L4**	1 (1.9%)	2 (2.8%)		0 (0.0%)	1 (3.1%)	
**Behavior, n (%)**			0.968			0.822
**B1**	26 (49.1%)	38 (51.4%)		5 (50.0%)	14 (43.8%)	
**B2**	16 (30.2%)	22 (29.7%)		5 (50.0%)	17 (53.1%)	
**B3**	4 (7.5%)	4 (5.4%)		0 (0.0%)	0 (0.0%)	
**B2+B3**	7 (13.2%)	10 (13.5%)		0 (0.0%)	1 (3.1%)	
**Combined perianal fistulas, n (%)**			0.217			0.477
**Yes**	34 (64.2%)	55 (74.3%)		6 (60.0%)	13 (40.6%)	
**No**	19 (35.8%)	34 (64.2%)		4 (40.0%)	19 (59.4%)	
**History of gastrointestinal surgery, n (%)**			0.753			0.875
**Yes**	25 (47.2%)	37 (50.0%)		5 (50.0%)	13 (40.6%)	
**No**	28 (52.8%)	44 (50.0%)		5 (50.0%)	19 (59.4%)	

Note: Mean ± SD. Abbreviations: CD, Crohn’s disease; BMI, body mass index; PhA, phase angle; WBC, white blood cell; HGB: hemoglobin; PLT, platelet; ALB, albumin; FIB, fibrinogen; ESR, erythrocyte sedimentation rate; CRP, C-reactive protein. *: *p* < 0.

**Table 3:** Logistic regression analysis of predictors of malnutrition based on GLIM criteria in men.

	OR	95% CI	*p*-Value
**Age (years)**	0.953	0.907–1.002	0.058
**PhA (°)**	0.150	0.062–0.362	0.000 *
**HGB (g/L)**	0.985	0.963–1.007	0.186
**ALB (g/L)**	0.977	0.869–1.099	0.700
**FIB (g/L)**	1.236	0.611–2.499	0.556
**ESR (mm/h)**	0.997	0.972–1.023	0.824
**CRP (mg/L)**	1.001	0.970–1.032	0.970
**Disease activity, n (%)**			
**Remission**	-	-	-
**Activity**	2.178	0.698–6.800	0.180

Note: Mean ± SD. Abbreviations: PhA, phase angle; HGB: hemoglobin; ALB, albumin; FIB, fibrinogen; ESR, erythrocyte sedimentation rate; CRP, C-reactive protein. *: *p* < 0.05.

**Table 4:** Comparison of patient characteristics, body composition, and clinical data between moderate malnutrition and severe malnutrition in CD.

	Male Malnutrition (n = 74)		*p*-Value	Female Malnutrition (n = 32)		*p*-Value
	Moderate Malnutrition (n = 40)	Severe Malnutrition (n = 34)		Moderate Malnutrition (n = 10)	Severe Malnutrition (n = 22)	
**Age (year)**	29.22 ± 9.90	29.15 ± 12.38	0.398	30.70 ± 15.88	28.36 ± 8.45	0.682
**BMI (kg/m^2^)**	19.23 ± 1.69	16.88 ± 2.33	0.000 *	19.28 ± 1.30	16.95 ± 1.87	0.001 *
**PhA (°)**	5.96 ± 0.59	5.39 ± 0.76	0.001 *	5.76 ± 0.90	4.90 ± 0.64	0.016 *
**WBC (** **×10^9^/L)**	5.94 ± 2.17	6.59 ± 2.45	0.278	6.20 ± 1.50	5.60 ± 2.05	0.222
**HGB (g/L)**	131.28 ± 21.99	118.44 ± 20.11	0.002 *	118.20 ± 10.65	102.68 ± 18.47	0.015 *
**PLT (** **×10^9^/L)**	295.75 ± 101.98	338.88 ± 107.55	0.045 *	259.90 ± 93.49	364.41 ± 133.35	0.034 *
**ALB (g/L)**	42.59 ± 4.89	37.34 ± 5.88	0.000 *	42.00 ± 4.35	36.98 ± 7.17	0.024 *
**FIB (g/L)**	3.51 ± 1.37	4.64 ± 1.35	0.002 *	3.32 ± 0.88	4.10 ± 1.31	0.074
**ESR (mm/h)**	32.97 ± 27.66	60.62 ± 32.35	0.000 *	44.80 ± 35.98	58.00 ± 37.68	0.299
**CRP (mg/L)**	17.84 ± 22.73	31.01 ± 28.05	0.011 *	7.22 ± 7.67	30.59 ± 33.97	0.074
**Disease activity, n (%)**			0.003 *			0.325
**Activity**	11 (27.5%)	21 (61.8%)		2 (20.0%)	10 (45.5%)	
**Remission**	29 (72.5%)	13 (38.2%)		8 (80.0%)	12 (54.5%)	
**Age of diagnosis, n (%)**			0.205			0.041 *
**A1**	3 (7.5%)	5 (14.7%)		3 (30.0%)	1 (4.5%)	
**A2**	28 (70.0%)	17 (50.0%)		5 (50.0%)	20 (90.9%)	
**A3**	9 (22.5%)	12 (35.3%)		2 (20.0%)	1 (4.5%)	
**Location, n (%)**			0.759			0.457
**L1**	9 (22.5%)	5 (14.7%)		0 (0.0%)	2 (9.1%)	
**L2**	1 (2.5%)	2 (5.9%)		1 (10.0%)	1 (4.5%)	
**L3**	29 (72.5%)	26 (76.5%)		9 (90.0%)	18 (81.8%)	
**L4**	1 (2.5%)	1 (2.9%)		0 (0.0%)	1 (4.5%)	
**Behavior, n (%)**			0.338			0.106
**B1**	19 (47.5%)	19 (55.9%)		7 (70.0%)	7 (31.8%)	
**B2**	11 (27.5%)	11 (32.4%)		3 (30.0%)	14 (63.6%)	
**B3**	2 (5.0%)	2 (5.9%)		0 (0.0%)	0 (0.0%)	
**B2+B3**	8 (20.0%)	2 (5.9%)		0 (0.0%)	1 (4.5%)	
**Combined perianal fistulas, n (%)**			0.145			0.662
**Yes**	27 (67.5%)	28 (82.4%)		3 (30.0%)	10 (45.5%)	
**No**	13 (32.5%)	6 (17.6%)		7 (70.0%)	12 (54.5%)	
**History of gastrointestinal surgery, n (%)**			0.641			1.000
**Yes**	21 (52.5%)	16 (47.1%)		4 (40.0%)	9 (40.9%)	
**No**	19 (47.5%)	18 (52.9%)		6 (60.0%)	13 (59.1%)	

Note: Mean ± SD. Abbreviations: CD, Crohn’s disease; BMI, body mass index; PhA, phase angle; WBC, white blood cell; HGB: hemoglobin; PLT, platelet; ALB, albumin; FIB, fibrinogen; ESR, erythrocyte sedimentation rate; CRP, C-reactive protein; A1: below 16 years old; A2: between 17 and 40 years old; A3: the age of diagnosis is above 40 years old; L1: ileal; L2: colonic; L3: ileocolonic; L4: isolated upper disease; B1: non-stricturing, non-penetrating; B2: stricturing; B3: penetrating; B2+B3: structuring and penetrating. *: *p* < 0.05.

**Table 5:** Logistic regression analysis of predictors’ degree of malnutrition based on GLIM criteria in men.

	OR	95%CI	*p*-Value
**PhA (°)**	0.246	0.079–0.767	0.016 *
**HGB (g/L)**	0.965	0.918–1.016	0.173
**PLT (** **×10^9^/L)**	0.994	0.985–1.004	0.245
**ALB (g/L)**	0.951	0.809–1.117	0.540
**FIB (g/L)**	2.362	0.952–5.861	0.064
**ESR (mm/h)**	1.019	0.981–1.058	0.329
**CRP (mg/L)**	0.965	0.921–1.012	0.142
**Disease activity**			
**Remission**	-	-	-
**Activity**	0.602	0.123–2.954	0.532

Note: Mean ± SD. Abbreviations: PhA, phase angle; HGB: hemoglobin; PLT, platelet; ALB, albumin; FIB, fibrinogen; ESR, erythrocyte sedimentation rate; CRP, C-reactive protein. *: *p* < 0.05.

**Table 6:** Logistic regression analysis of predictors’ degree of malnutrition based on GLIM criteria in women.

	OR	95%CI	*p*-Value
**PhA (°)**	0.000	0.000–0.627	0.037 *
**HGB (g/L)**	0.855	0.702–1.042	0.855
**PLT (** **×** **10^9^/L)**	1.026	0.992–1.060	0.133
**ALB (g/L)**	0.771	0.482–1.232	0.771
**Age of diagnosis**			
**A1**	-	-	-
**A2**	728.109	0.008–62604377.3	0.172
**A3**	1.026	0.992–1.060	0.133

Note: Mean ± SD. Abbreviations: PhA, phase angle; HGB: hemoglobin; PLT, platelet; ALB, albumin; A1: below 16 years old; A2: between 17 and 40 years old; A3: the age of diagnosis is above 40 years old. *: *p* < 0.05.

## Data Availability

All data included in this study are available upon request by contact with the corresponding author.
